# Supplementary figures and images for: Global mistranslation increases cell survival under stress in Escherichia coli
Source: PLoS Genet. 2020 Mar 9;16(3):e1008654. doi: 10.1371/journal.pgen.1008654 (PMC7082066; doi:10.1371/journal.pgen.1008654)

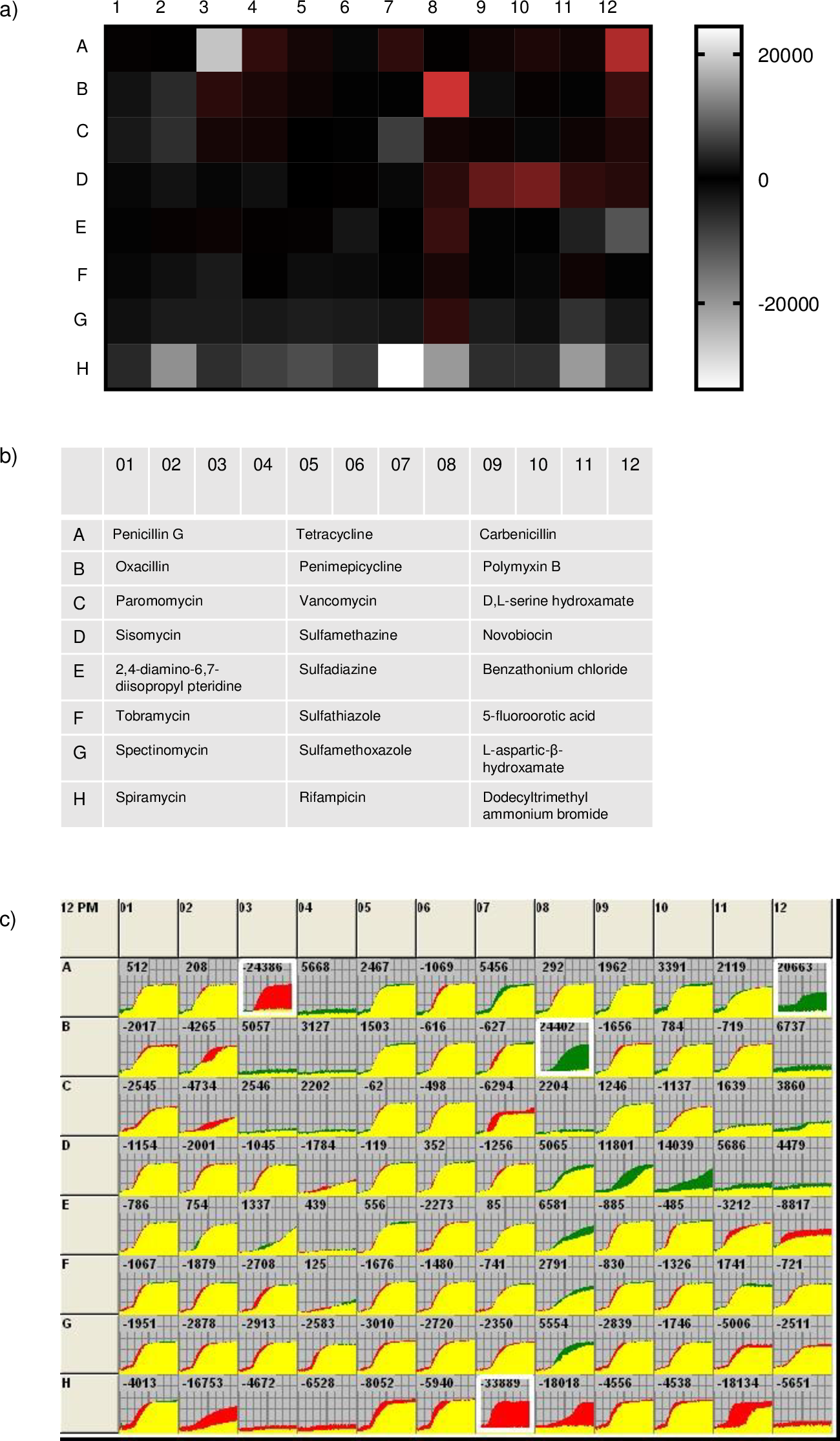

Supplement: S1 Fig — (a) WT and Mutant (n = 1 for one plate) were inoculated in the phenotype micro-array antibiotic plate PM12B. Differences in the area under the curve of growth (AUC) for Mutant-WT after 48h are shown here in a heat map. Each of the 24 antibiotics is present in 4 wells, with 2X increasing concentrations in successive wells. Absolute concentrations are proprietary and unknown. (b) Plate showing the antibiotics used. (c) Expanded version showing raw growth curves as obtained from the software, with the arbitrary numbers indicating dye reduction values. Red shows WT dye reduction, green, Mutant, and yellow shows the region of overlap. The four highest values are highlighted automatically by the software (white rectangles). (TIF) [file pgen.1008654.s002.tif]

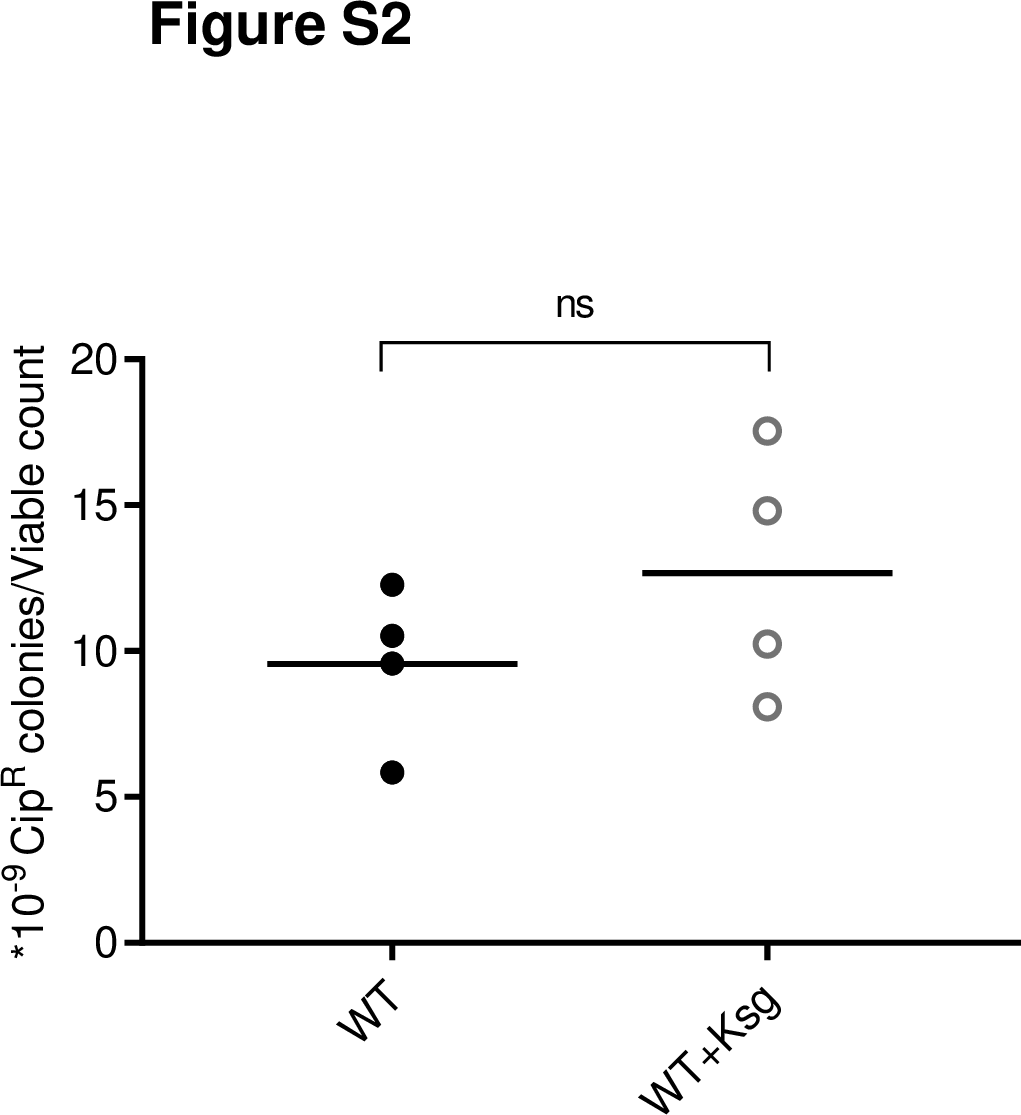

Supplement: S2 Fig — Survival of WT and WT+Kasugamycin (Ksg) cultures from single colonies (n = 4) pulsed with 20 ng/ml ciprofloxacin (Cip) for 1 hr and then plated on LB plates with vs. without 50 ng/ml Cip (Cip50). Plot shows the number of resistant colonies per unit viable count from LB plates, means are indicated. Mann-Whitney U test, WT vs WT (Ksg), ns, U = 5, P = 0.49 (TIF) [file pgen.1008654.s003.tif]

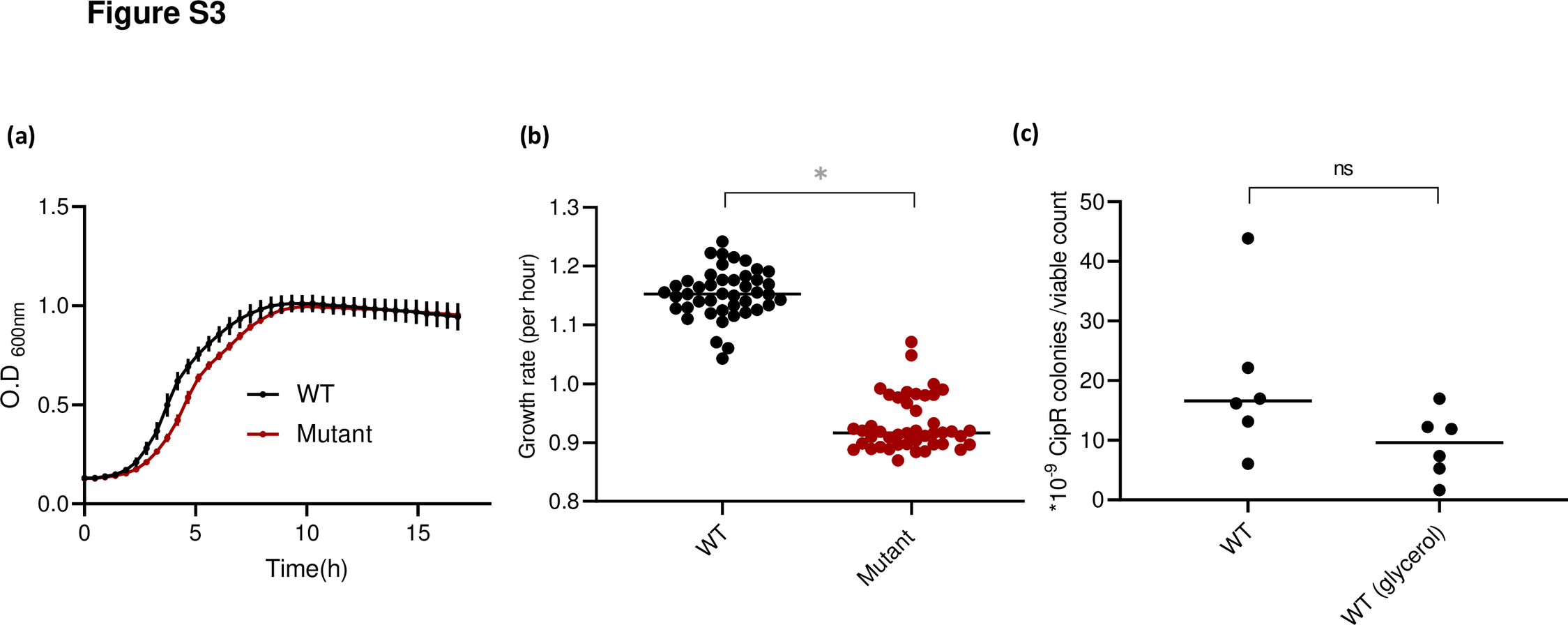

Supplement: S3 Fig — (a) Raw growth curves for WT and Mutant (n = 40) showing mean +/- SD values as obtained by a Tecan growth reader recording OD600 every 30 minutes ((b) Mutant grows slower than the WT. WT and Mutant (n = 44) doubling times estimated from the growth curve, means are indicated. Mann-Whitney U test, WT >Mutant, U = 4, P<0.0001 (c) Reducing WT growth rate does not impact ciprofloxacin resistance. Survival of WT and Mutant cultures from single colonies (n = 6) pulsed with 20 ng/ml ciprofloxacin (Cip) for 1 hr and then plated on LB plates with vs. without 50 ng/ml Cip (Cip50). Plot shows the number of resistant colonies per unit viable count from LB plates, means are indicated. Mann-Whitney U test, WT vs WT (glycerol), ns, U = 1, P = 0.07. (TIF) [file pgen.1008654.s004.tif]

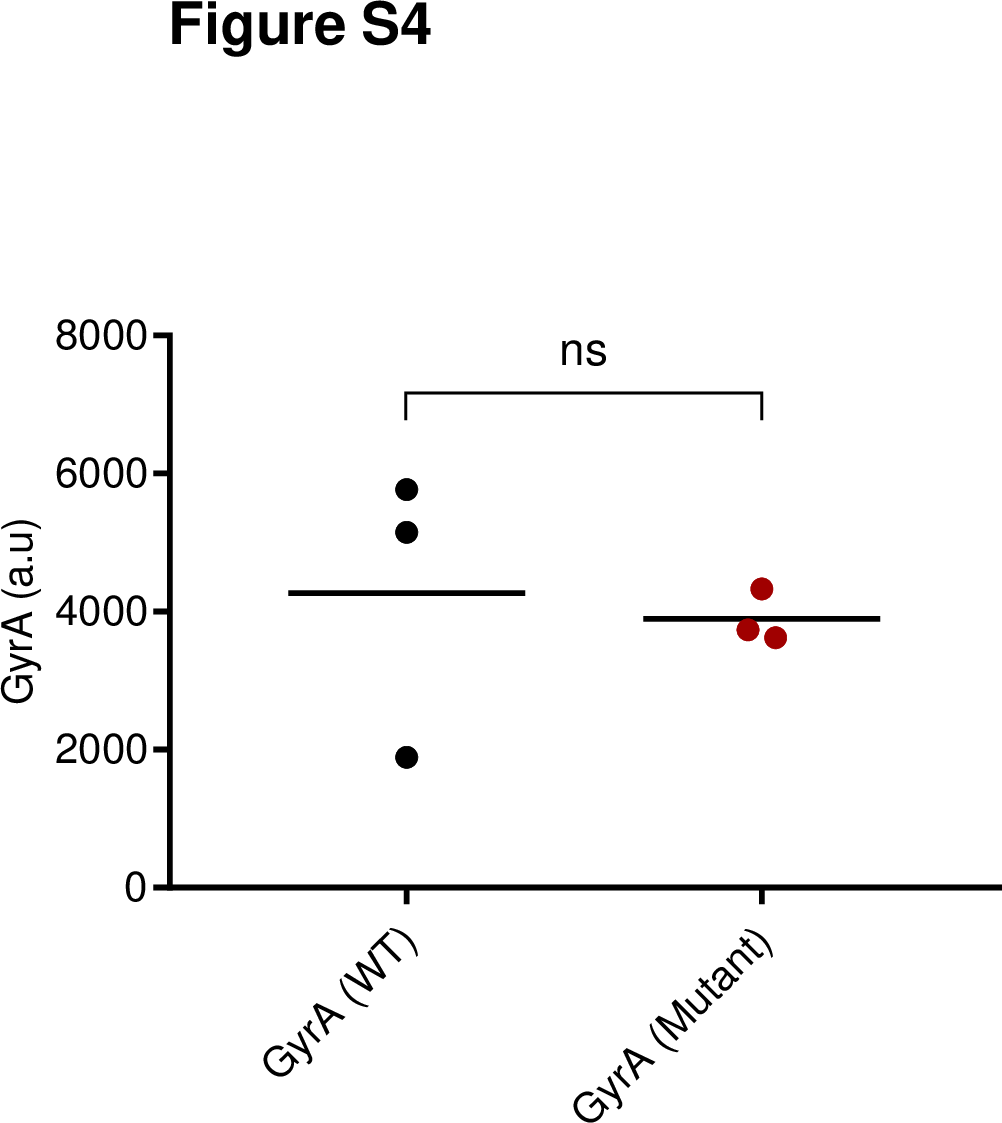

Supplement: S4 Fig — Cell extracts from WT and Mutant (n = 3) were used to carry out western blotting for GyrA. Means are indicated. Unpaired t test, WT vs Mutant, ns, t = 0.305, P = 0.76 (TIF) [file pgen.1008654.s005.tif]

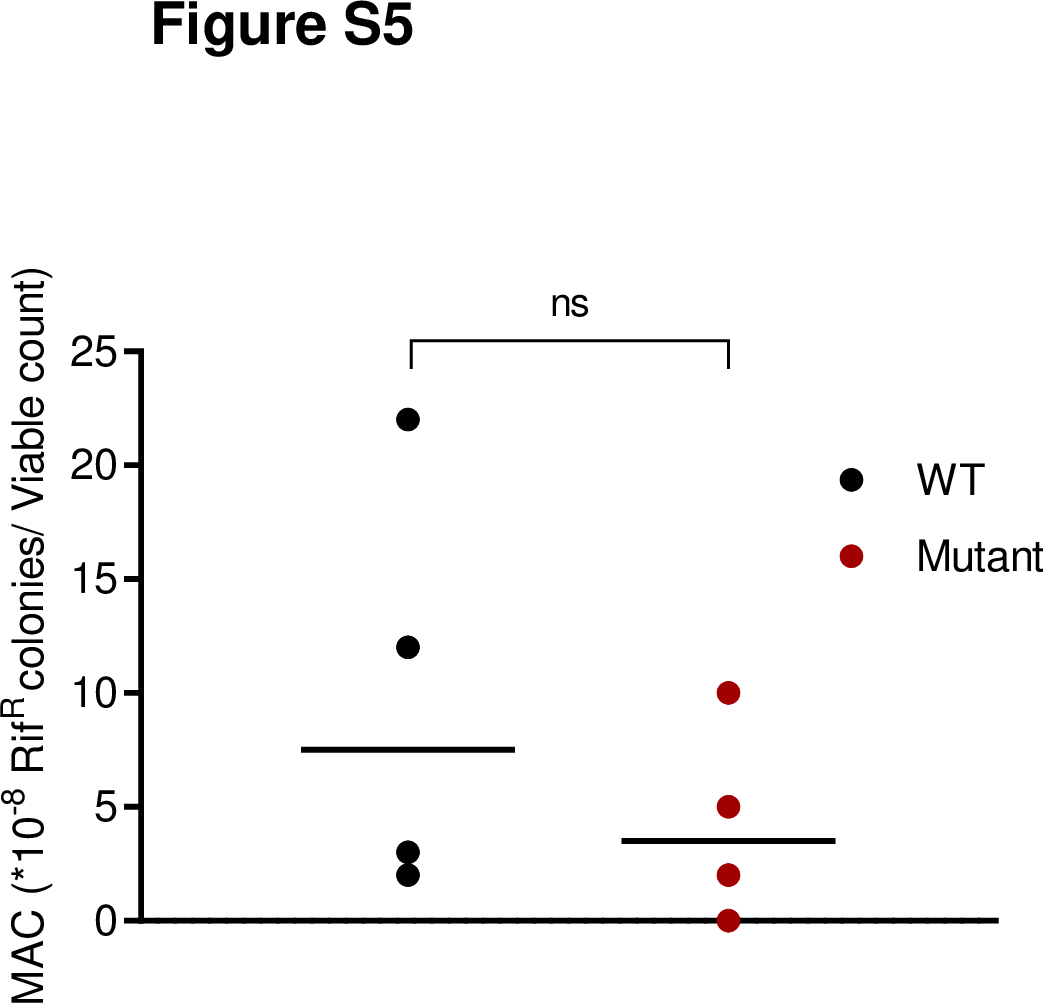

Supplement: S5 Fig — ~1000 cells each from mid-log phase cultures of WT and Mutant (n = 4) were plated onto Rif-50 agar plates and incubated for 5 days. Plot shows the number of rifampicin resistant colonies per unit viable count after this period, means are indicated. Mann-Whitney U test, WT vs Mutant, ns, U = 13.5, P = 0.17 (TIF) [file pgen.1008654.s006.tif]

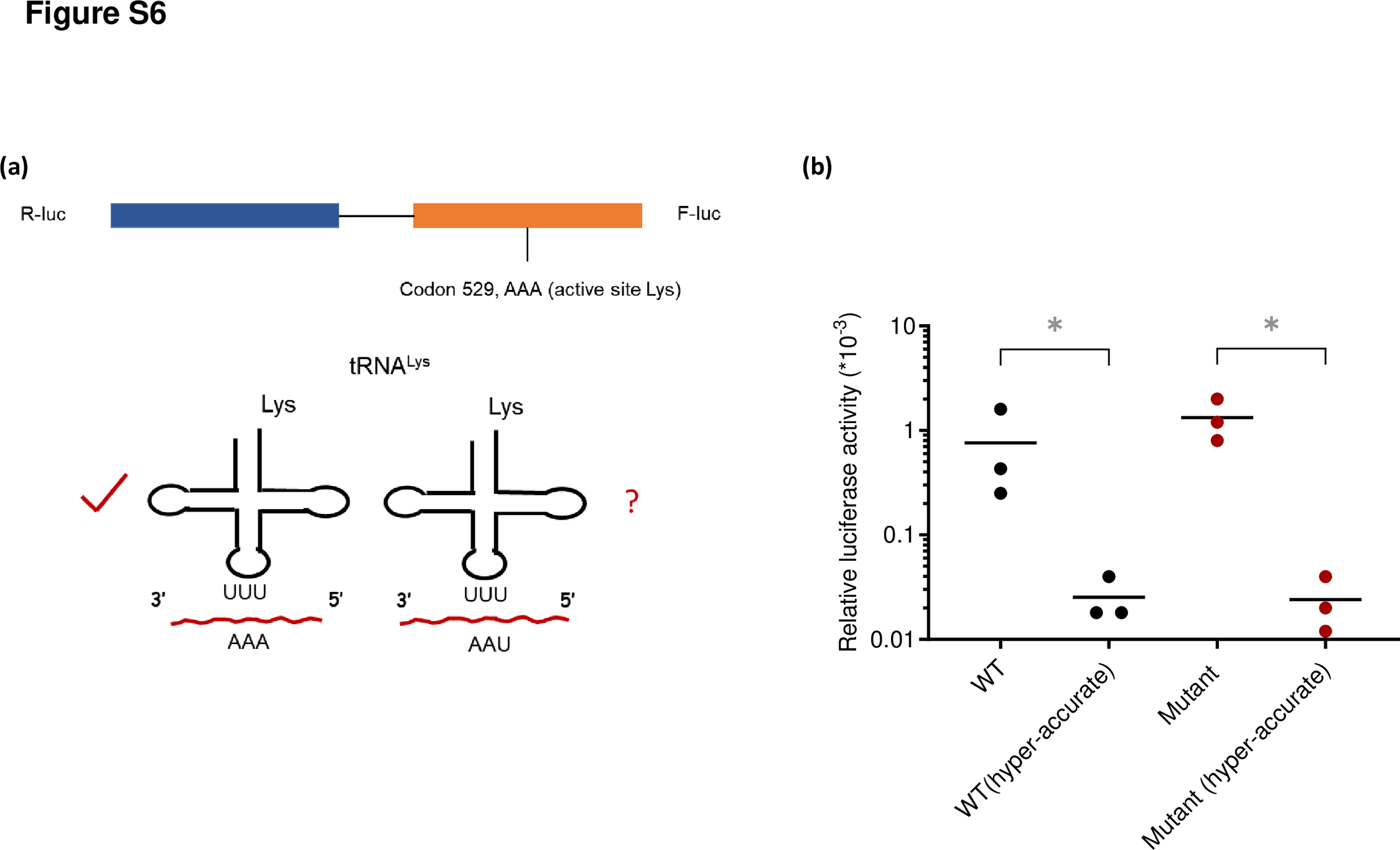

Supplement: S6 Fig — (a) Schematic showing the Renilla (R-luc) and Firefly (F-luc) luciferase genes along with the correct and incorrect (mistranslating) codon recognition by tRNALys (b) Mean mistranslation rates measured with an in vitro dual luciferase assay for WT and Mutant (n = 3). Paired t tests, WT (hyper-accurate)<WT, t = 6.9, P = 0.006, Mutant (hyper-accurate)<Mutant, t = 4.8, P = 0.008) (TIF) [file pgen.1008654.s007.tif]

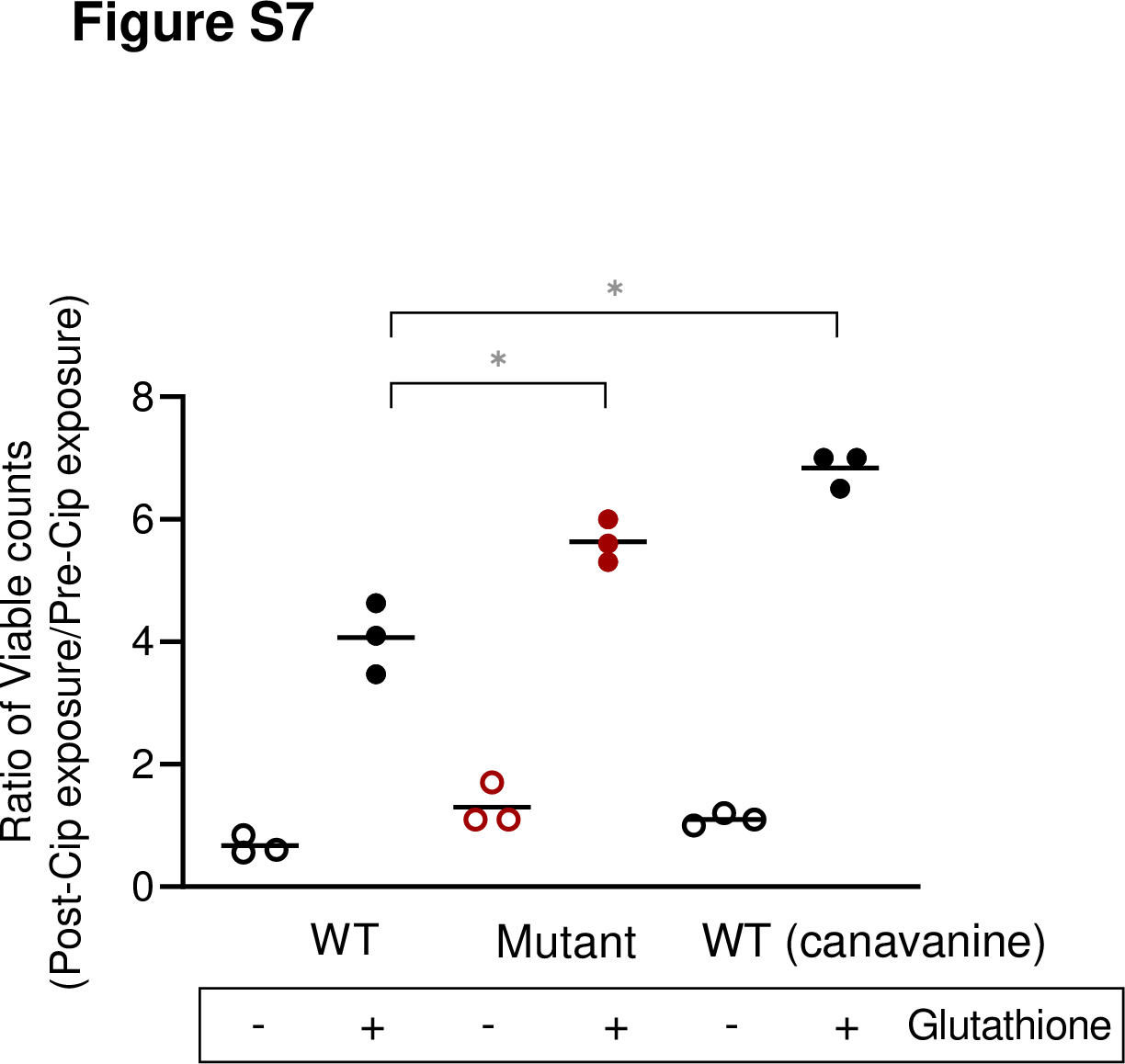

Supplement: S7 Fig — Survival of midlog phase cultures (OD600nm~0.6) of indicated strains, inoculated from single colonies (n = 3) treated with 50 ng/mL Cip for 2h and then plated on LB agar. The plot shows the average ratio of number of colonies before and after Cip treatment. Unpaired t tests with glutathione treatment: Mutant>WT, t = 3.9, P = 0.02; WT(canavanine)>WT, t = 7.5, P = 0.005. (TIF) [file pgen.1008654.s008.tif]

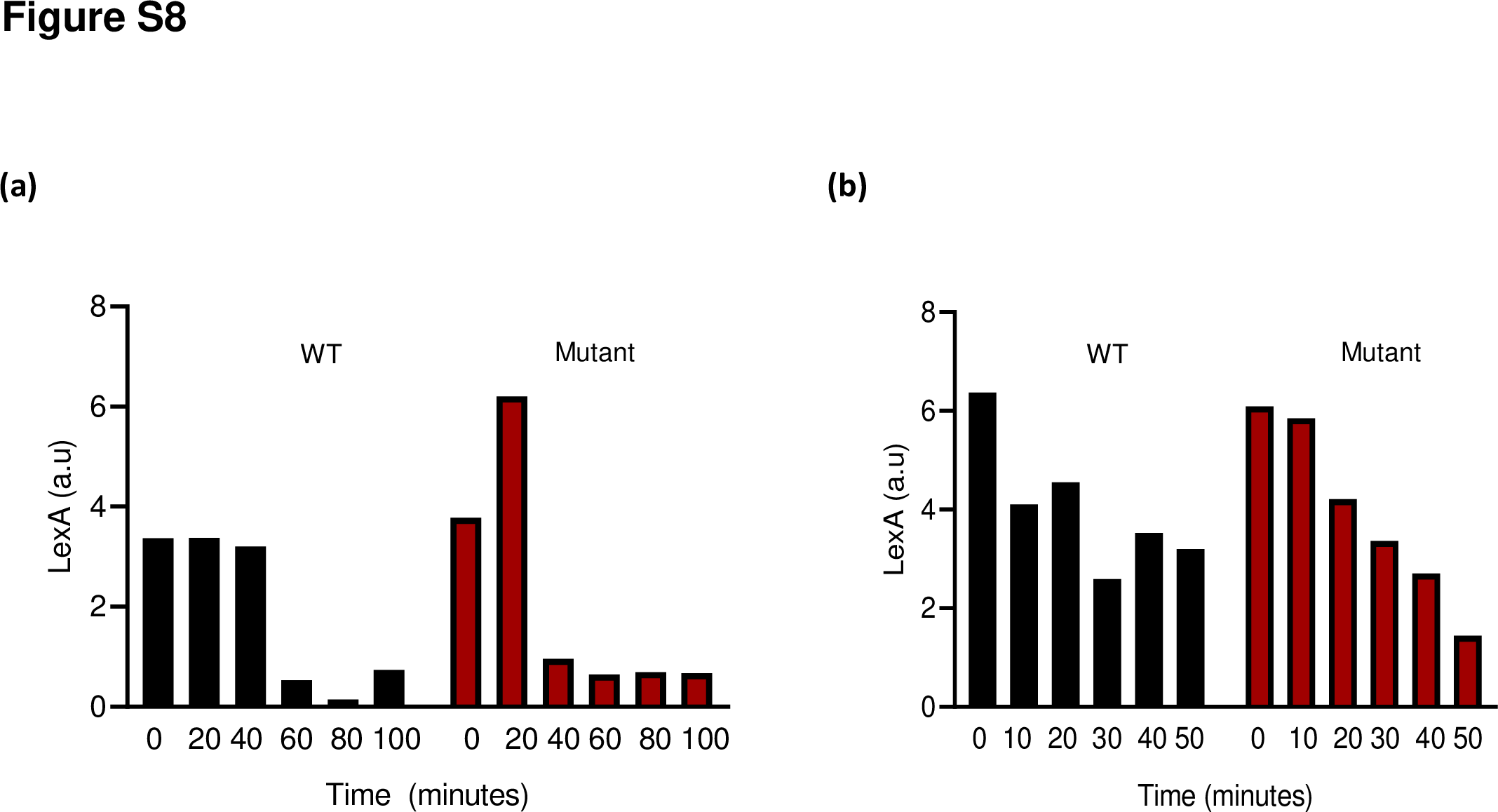

Supplement: S8 Fig — LexA protein levels normalised to total protein as measured by western blotting using a polyclonal anti-LexA antibody. Each panel shows data for an independent experimental block, in addition to the block shown in Fig 2E. (TIF) [file pgen.1008654.s009.tif]

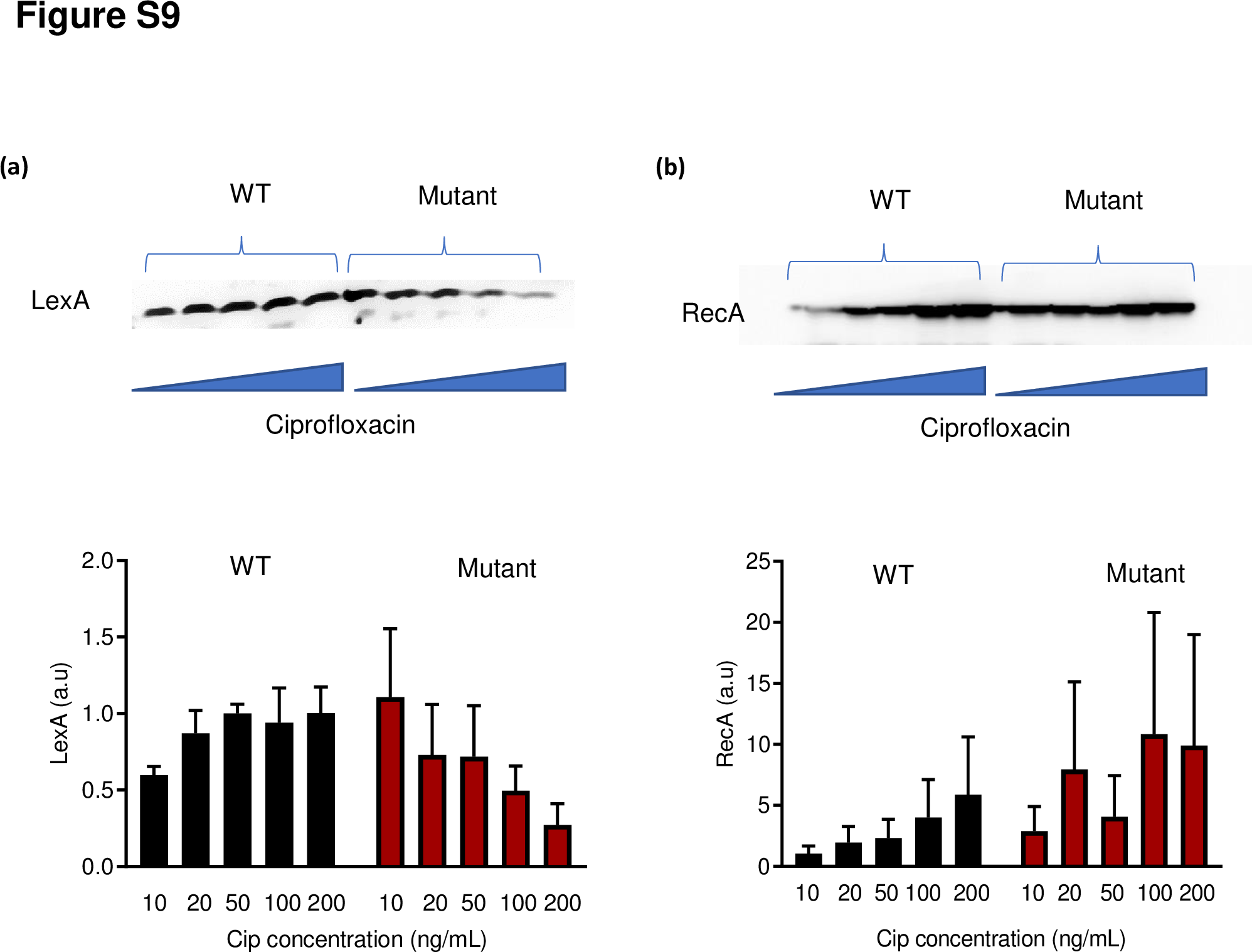

Supplement: S9 Fig — (a) LexA protein levels in WT and mutant cultures exposed to different Cip concentrations for 30 mins, measured by western blotting using a polyclonal anti-LexA antibody. Quantitation across biological replicates (n = 3) is shown (mean±SD). (b) RecA protein levels in WT and mutant cultures, measured by western blotting using a polyclonal anti-RecA antibody. Quantitation across biological replicates (n = 3) is shown (mean±SD). (TIF) [file pgen.1008654.s010.tif]

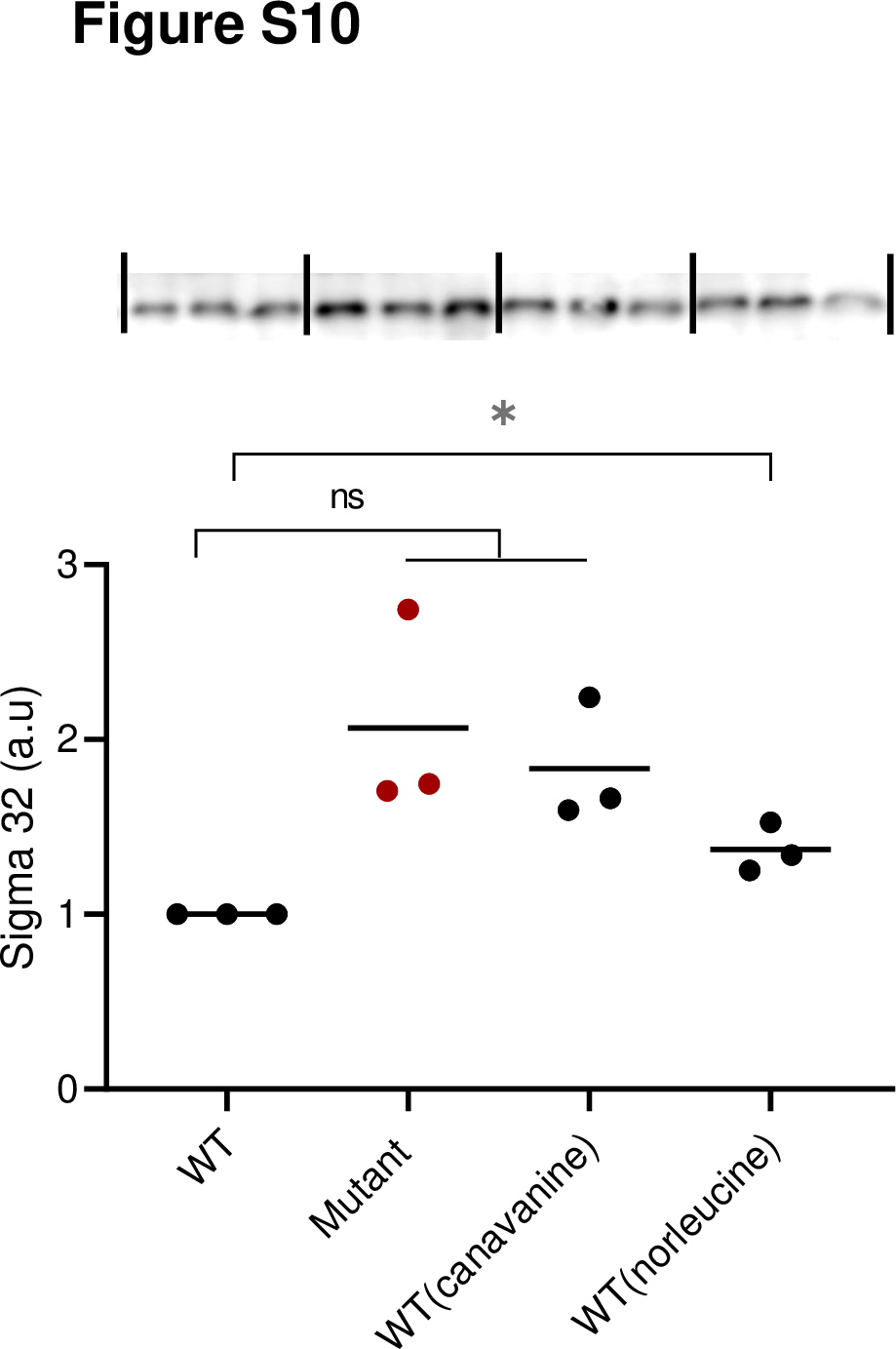

Supplement: S10 Fig — Mean levels of Sigma 32 protein in mid log phase cultures (OD600~0.6) assessed by western blotting using a polyclonal anti-sigma 32 antibody. Quantitation across biological replicates (n = 3) is shown. Paired t tests: Mutant vs. WT, ns, t = 3.1, P = 0.08; WT (canavanine) vs. WT, ns, t = 4.08, P = 0.05; WT(norleucine)>WT, t = 4.5, P = 0.04. (TIF) [file pgen.1008654.s011.tif]

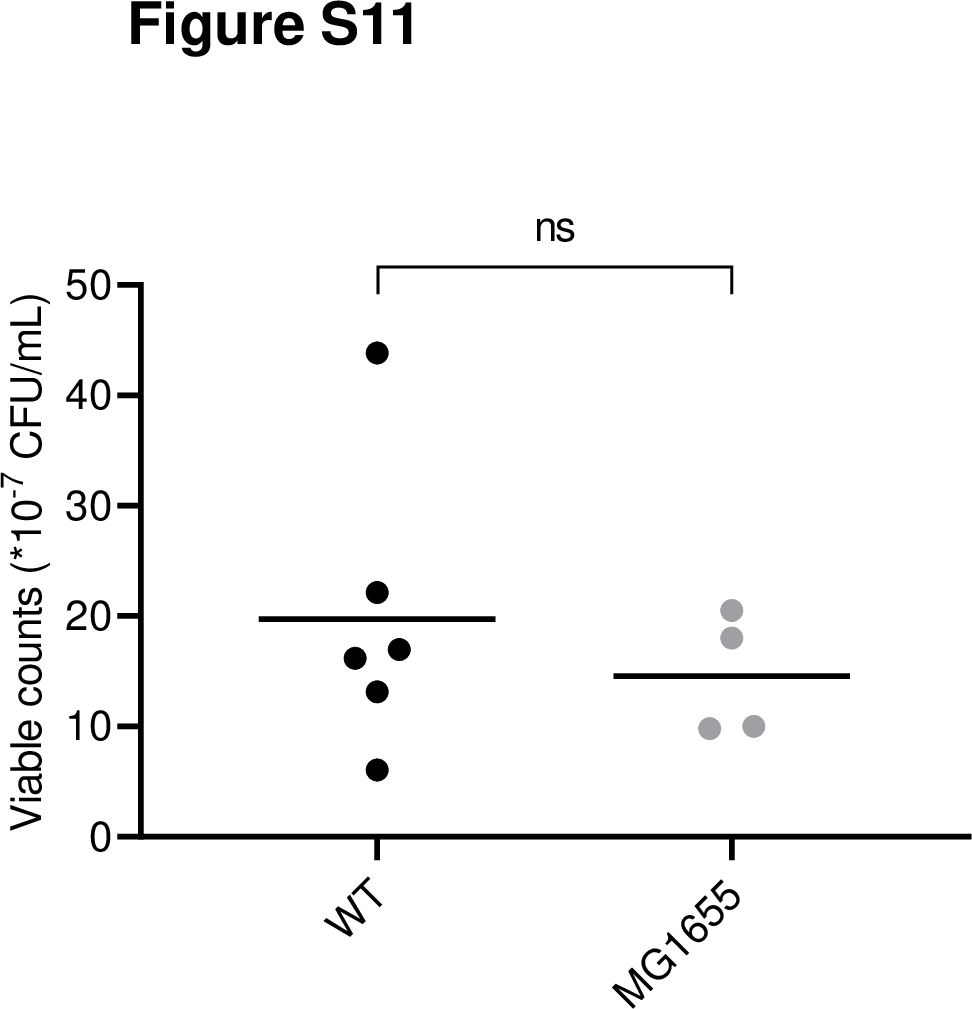

Supplement: S11 Fig — Resistance of mid log phase cultures of WT (KL16) and MG1655 (OD600nm~0.6) from single colonies (n = 6) pulsed with Cip 20 for 1 h and plated on LB agar with vs. without Cip 50. Plot shows the mean number of resistant colonies per unit viable count from LB agar plates. Mann-Whitney U test, WT vs MG1655, ns, U = 10, P = 0.76. (TIF) [file pgen.1008654.s012.tif]

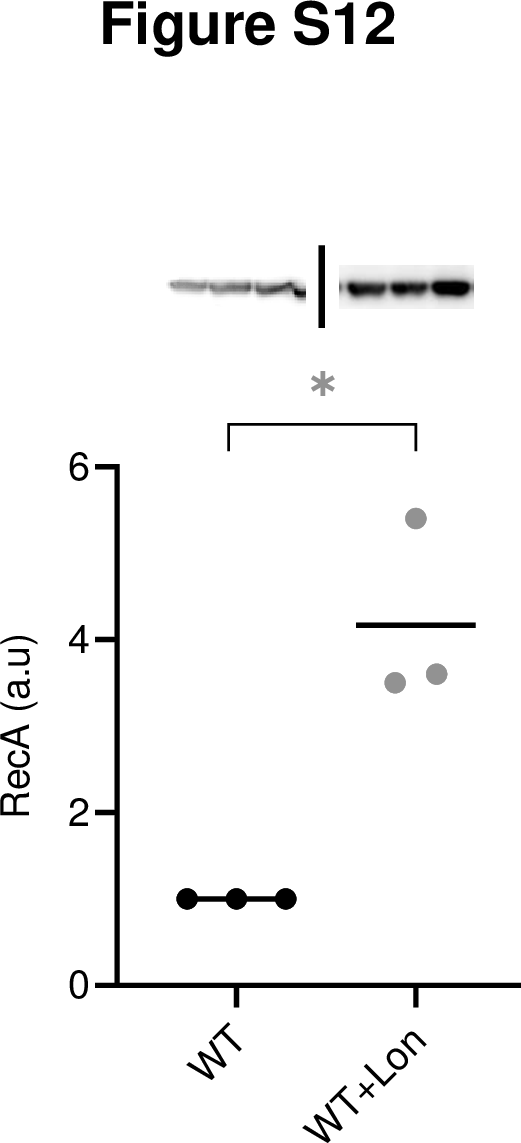

Supplement: S12 Fig — RecA protein levels in mid log phase (OD600~0.6) cultures assessed by western blotting using a polyclonal anti-RecA antibody, normalised to total protein. The figure shows bands from two separate blots.t test: WT+Lon>WT, t = 5.13, P = 0.03. (TIF) [file pgen.1008654.s013.tif]

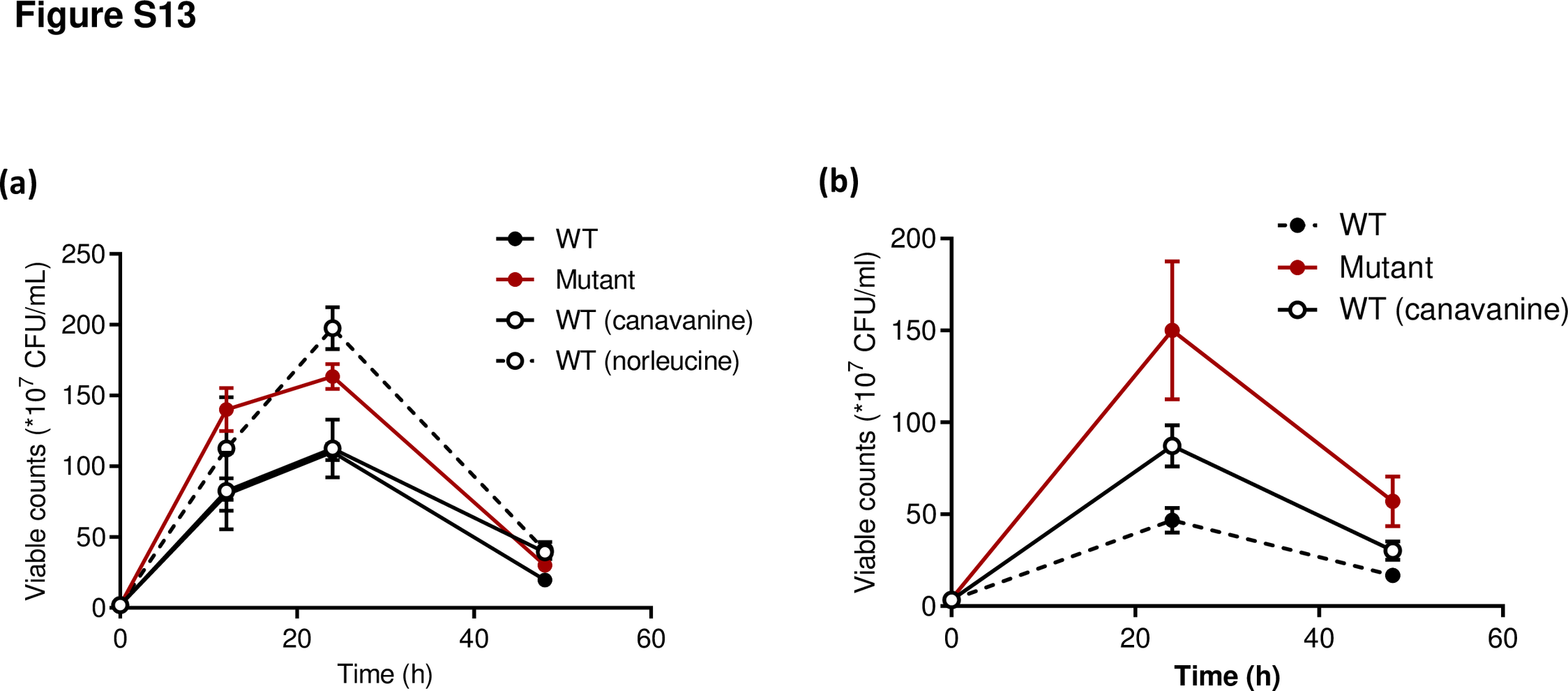

Supplement: S13 Fig — Survival of cultures inoculated from single colonies (n = 3), dilution plated at indicated times on LB agar. Two experimental blocks are shown. Plot shows the total viable counts (mean±SEM). (a) t-tests at 24 h: Mutant>WT, t = 7.2, P<0.001; WT(norleucine)>WT, t = 5.6, P = 0.007; WT vs WT(canavanine), ns, t = 0.12, P = 0.9 (b) t-tests at 24 h: WT vs Mutant, ns, t = 2.8, P = 0.06; WT(canavanine)>WT, t = 3.1, P = 0.02. (TIF) [file pgen.1008654.s014.tif]

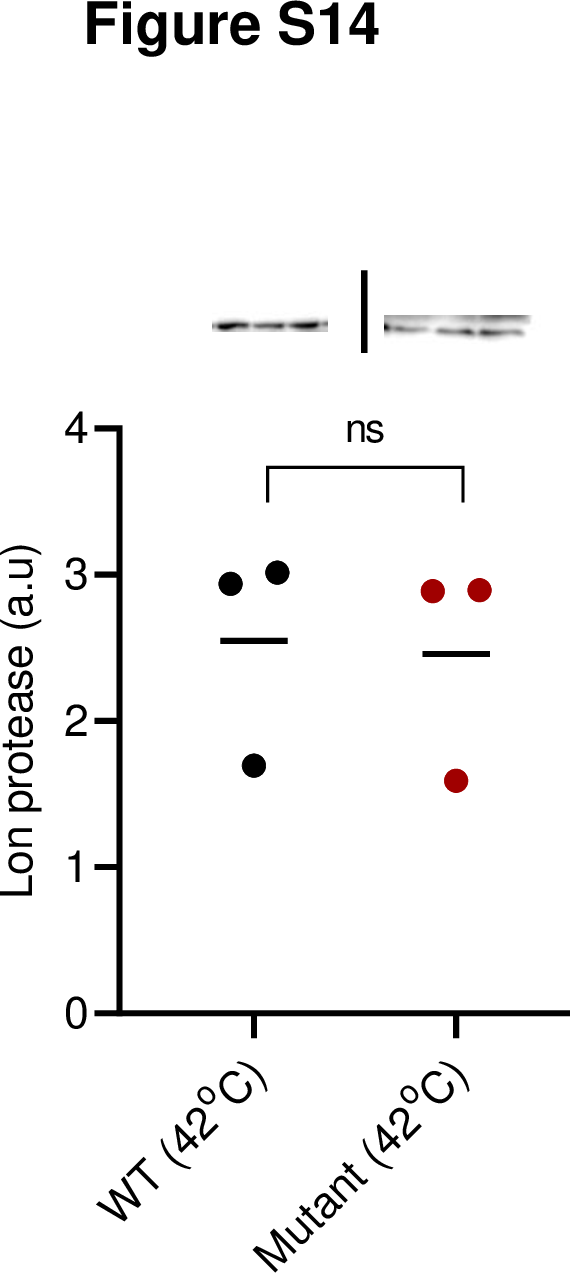

Supplement: S14 Fig — Mean levels of Lon protein in mid log phase (OD600~0.6) cultures of WT and Mutant (n = 3) as measured by western blotting using a polyclonal anti-Lon antibody, normalised to total protein. Paired t test: WT vs Mutant, ns, t = 0.9, P = 0.12. (TIF) [file pgen.1008654.s015.tif]

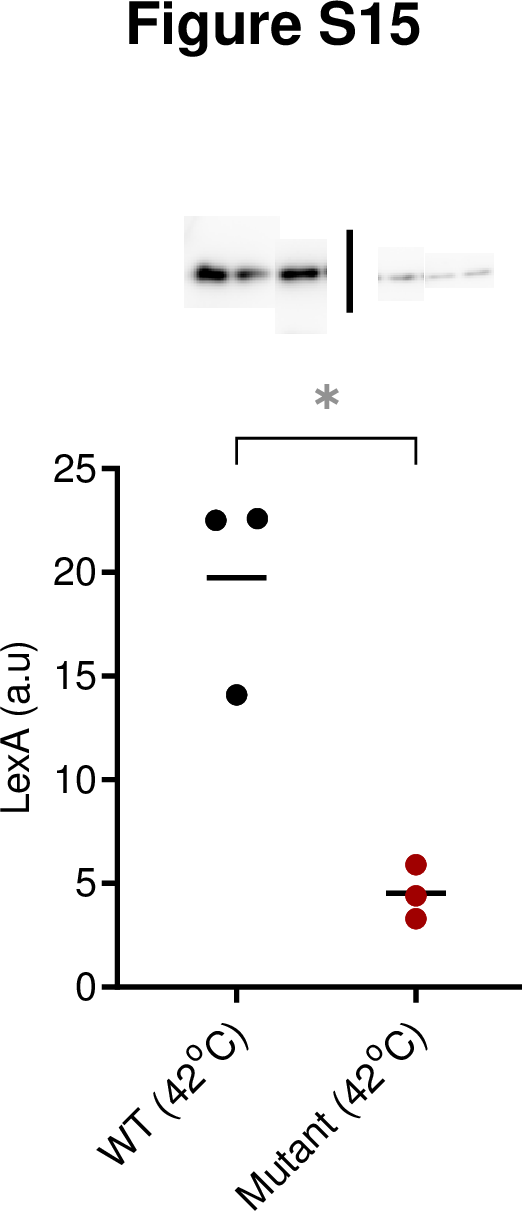

Supplement: S15 Fig — Mean LexA protein levels in mid log phase (OD600~0.6) cultures of WT and Mutant (n = 3) as measured by western blotting using a polyclonal anti-Lon antibody, normalised to total protein. Paired t test: Mutant<WT, t = 5.3, P = 0.03. (TIF) [file pgen.1008654.s016.tif]

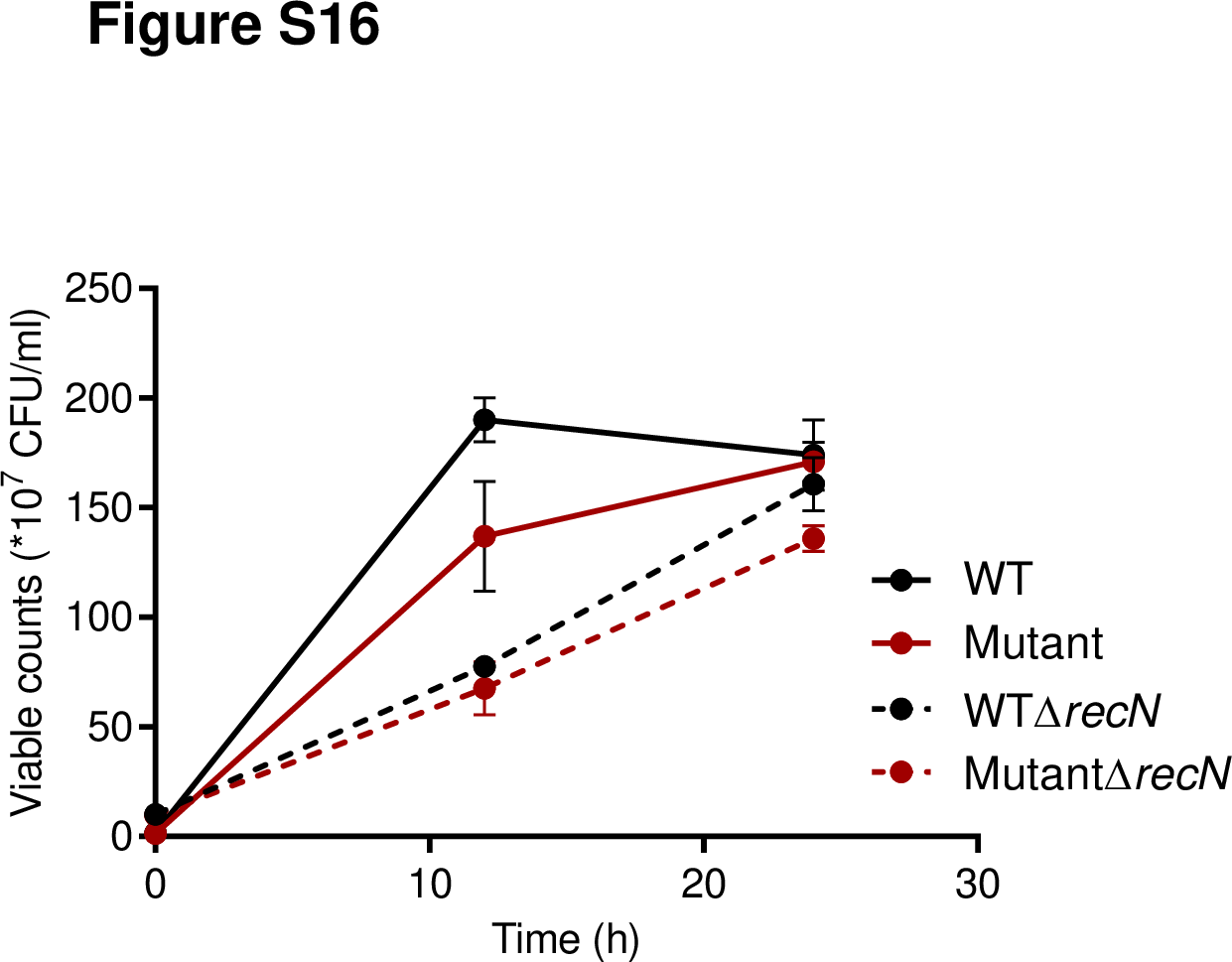

Supplement: S16 Fig — Survival of cultures inoculated from single colonies (n = 3 for WTΔrecN and MutantΔrecN and n = 5 for WT and Mutant), dilution plated at indicated times on LB agar. Plot shows the total viable counts (means±SEM). Unpaired t-tests at 12 h: WTΔrecN<WT, t = 17.8, P<0.0001; MutantΔrecN<Mutant, t = 5.2, P = 0.002. At 24 h: WTΔrecN vs. WT, ns t = 0.79, P = 0.28; MutantΔrecN <Mutant, t = 5.2, P = 0.002. (TIF) [file pgen.1008654.s017.tif]
