# Supplementary material for: Global mistranslation increases cell survival under stress in Escherichia coli
Source: PLoS Genet. 2020 Mar 9;16(3):e1008654. doi: 10.1371/journal.pgen.1008654 (PMC7082066; doi:10.1371/journal.pgen.1008654)
Supplement: Attachment — Submitted filename: Response to reviewers.docx [file pgen.1008654.s018.docx]

**Reviewer #1**

This paper shows that increasing the global rate of mistranslation (in one of a couple of different ways) leads to increased cell survival in several stressful environments due to upregulation of the SOS response. More stress-resistance mutants appear not through mutagenesis but through higher survival yielding more opportunities for mutation. I am not expert in the experimental techniques nor molecular biology details, but it looks sound to me, as do the statistics. I find the manuscript to be novel, important, and convincing.

My only comments/suggestions concern some details of the conceptual framing, primarily in the Introduction. The Introduction lists hypotheses for why mistranslations have evolved to be high, as the question motivating the paper:
1) Speed-accuracy tradeoff
2) Robustness to consequences of error may weaken selection against making them (which is a special case of a broader drift barrier hypothesis that selection isn’t strong enough to care)
3) Bet-hedging advantages of a statistical proteome
4) The proposed new hypothesis of inducing stress responses

An important distinction that the current presentation blurs is that 1) and 2) are hypotheses for the evolution of baseline mistranslation rates, while 4) predicts the evolution of mistranslation rates that rise in response to stress. As currently written, 3) can be read either way, although I think it is a stronger hypothesis if it is with respect to induced high rates. Given that they are evolutionary hypotheses for the causes of somewhat different things, I suggest writing 1) and 2) in one paragraph that is more of a preamble rather than part of a direct numbered contrast to 4), then segueing to inducible vs constitutively high mistranslation rates as part of introducing 3) as real and currently popular hypothesis. Distinguishing between baseline and induced mistranslation rates makes 4) an alternative to 3), but not to 1) and 2) as they are currently written. There are null alternatives re induced rates that are related to 1) and/or 2) but not quite identical, eg that cells in trouble ditch proofreading because they have other stuff to worry about (eg speed in responding to the stress) or that selection is not strong enough to make high fidelity stress-proof, and I think it would be fine to introduce those if made distinct from baseline rates.

Coming back to this framework after the results are presented, what has been shown by the experiments is that it would be beneficial to have higher stress-induced mistranslation rates than those that have in fact evolved. So rather than answer the puzzle of why they have evolved so high, as currently framed in the Introduction, we are left with a puzzle of why they didn’t evolve higher! I suggest acknowledging and maybe speculating about this new puzzle in the Discussion.

Signed,
Joanna Masel

**Response:** We thank Dr Masel for her insightful input. She raises two key points:

Separating hypothesis 1) and 2) from 3) and 4), as being relevant to the evolution of basal vs. induced mistranslation rate. We agree that this is a useful distinction. However, hypotheses 3) and 4) are also applicable to the evolution of baseline mistranslation rates, under the right circumstances (e.g. under a high rate of encountering stress, a high baseline error rate may be more beneficial than induced mistranslation). We have now included this point while introducing hypothesis 3, and framed our hypothesis accordingly (Page 3, Lines 55-58).

The second point refers to why we do not see even higher mistranslation rates. We can only speculate, but one definite limiting factor is the cost of mistranslation under non-stressful conditions, which will determine how many cells with extreme mistranslation rates survive in a population. We expect that the nature and shape of the tradeoff between the cost and benefit of mistranslation under stress vs. normal conditions will depend on – presumably among other factors – the magnitude and frequency of stressful conditions. We now mention such a trade-off more clearly in the Introduction (lines 71-78. We have also added a new paragraph in the Discussion section, where we speculate further about this tradeoff. We also expand on the specific case of DNA damage as a commonly encountered stress where an SOS response – and potentially mistranslation – are more broadly relevant for cell survival (Page 12, lines 361-379).

**Reviewer #2**

This is potentially a very interesting paper. Samhita and et al., examined the effect of higher mistranslation on phenotypes of an E. coli strain and discovered that higher mistranslation rate can cause increase in resistance to a fluoroquinolone antibiotic that inhibits DNA gyrase. They also unveiled the mechanisms of cellular responses to high mistranslation underlying high resistance to fluoroquinolone antibiotics, e.g., SOS response. Subsequently, they showed that high mistranslation rate cause tolerance to high temperature. This is a nice piece of work that convincingly demonstrate that mistranslation trigger SOS response in E. coli, and increase tolerance and resistance to a fluoroquinolone antibiotic and high temperature. The findings presented in this work provide a new knowledge and interest to the audience in PLoS Genetics. However, I have few issues in the manuscript which should be addressed before it is published.

**Response:** We thank the reviewer for their encouraging comments.

**Comment 1:** First, I am very confused and frustrated by how the authors presented and discussed about their phenotypic microarray assays of E. coli (WT vs Mutant). Initially, they have done 48 conditions but did not describe what these antibiotics are (no name of antibiotics), but only highlighted three antibiotics that showed what they discussed in the manuscript. This experiment should provide much more meaningful information to this work and the authors should describe carefully in the manuscript and provide all experimental data and information in supplementary figure and table. I don’t see a reason why the authors ignored completely these antibiotics that exhibit negative effects on Mutant. What kind of antibiotics are more negative effects on Mutant over WT? It looks like that the majority of antibiotics are negative on Mutant. Should such negative effects on Mutant provide some information about how mistranslation alters phenotype of E. coli cells too? Can the authors rationalize these negative effects? The colour scheme of Supplementary Fig 1 should be changed, e.g., 0 (WT=Mutant should be white and one colour as positive and another as negative.

**Response:** We apologise for the confusion surrounding the Biolog phenotype microarrays. We did not discuss these results in detail because this was only intended to be a preliminary qualitative assay, to be verified in standard laboratory growth conditions before proceeding. All work reported in the paper follows from the laboratory assays for DNA damage shown in Fig.1.

The Biolog plate assays involve static growth for 48h, with only one biological replicate per plate (each replicate is exposed to four antibiotic concentrations, as is now shown in Fig.S1b). The readout is not directly of growth, but rather of respiration resulting in the reduction of the tetrazolium dye followed by a colour change. Therefore, it differs substantially from laboratory bacterial growth in aerated shaking cultures, where actual viable counts or optical density are measured. We found from prior experience (unpublished), as have others before (Leiby and Marx, PLoS Biology, 2014), that Biolog plates often show differences between two strains that cannot be replicated under laboratory conditions, and sometimes not even in a second run of the same plate. As a result, we only used these arrays as a preliminary screen for conditions that we should pursue further.

We do agree, however, that the Biolog data could be shown more clearly. We have now changed the colours in the heat map as suggested (Fig.S1a) and have added Fig.S1b to show the actual antibiotics used, and Fig.S1c to show the actual readout obtained from the Biolog assay software. As is perhaps clearer now, under most conditions, there is no difference between WT and Mutant (black colour, Fig.S1a). In three conditions, the Mutant performed better than the WT (red colour, Carbenicillin, Novobiocin and Penimipecycline); although only Novobiocin showed consistent results across different antibiotic concentrations. Hence, we used Novobiocin as a starting point to investigate DNA damaging antibiotics, and focused on the impact of ciprofloxacin (belonging to the same class of antibiotics) in detail, forming the basis for this paper. In five other antibiotics, WT does better than the Mutant (grey colour, Penicillin G, Benzathonium chloride, Spiramycin, Rifampicin and Dodecyl trimethyl ammonium bromide), but again this was generally inconsistent across concentrations, underscoring the problem with using these arrays as a direct readout for fitness. Of these, we verified that the Mutant is indeed poorer in Rifampicin.

In summary:

1. Most antibiotics do not differentially impact the Mutant vs the WT, i.e, mistranslation is neither beneficial nor deleterious in most cases

2. We have now provided all information including antibiotic names and raw dye reduction values

3. We have changed the colour scheme of the heatmap

4. We have only shown data for 24 antibiotics; we have corrected the erroneous mention of 48 conditions in the Introduction (Page 5, line 118).

5. We have only used this qualitative preliminary assay as a starting point to begin investigating the impact of DNA damage on mistranslation; we have not made any inferences from these data.

**Comment 2:** Related to the above, I feel that the argument that the author made in the manuscript “mistranslation can be adaptive trait” is overstating. The authors made a strong argument on this subject and generalize their observation e.g., page 10 “we demonstrate that mistranslation is generally beneficial under stress”. The authors only see tolerance to a fluoroquinolone antibiotic and high temperature, and ignored the fact that the most antibiotics cause negative effects on the high mistranslation strain in the manuscript, and it sounds that the authors form a theory based on very biased information that they like to see, and not based on comprehensive and unbiased data. Again, it is hard to discuss here as they did not present the microassay data properly (they should provide full description of their data in the microarray, and discussed the data extensively in the results and discussion), but they author should do more balanced discussion throughout results and discussion. Some recent papers demonstrated recently that (PMID:30936490, PMID: 27111525), genetic optimization of proteins can be understood only considering fitness in multiple environmental effects, but not only one environment. I presume that similar attribute applies to the organismal level. Seeing higher fitness in few environments does not mean that it is generally good, as bacteria are living environments that constantly changing and fluctuating. I suggest the authors to consider such aspect carefully and revise their discussion to more unbiased manner.

**Response:** We hope that the clarification about the Biolog arrays and the altered Fig.S1 show that mistranslation is actually neither deleterious nor beneficial in most antibiotics.

We did not intend to convey that mistranslation is beneficial under all kinds of stresses; thank you for pointing this out. Page 10, line 320 now reads ‘We demonstrate that mistranslation is generally beneficial under DNA damage’. The stress resistance benefits we present in this work are limited to benefits that come up through activation of the SOS response. However, the SOS response is increasingly turning out to be relevant not only for DNA damage, but also for several other stress resistance phenotypes including high temperature (as we show), but also high pressure and persistence to antibiotics (references included in the last paragraph of the discussion, Page 12, line 383-385). Hence, we believe that our discussion of a more generalised stress resistance phenotype is justified.

**Comment 3:** It is very unclear how many samples that the authors sequenced (whole genome sequencing) to determine mutations in the genome, and which mutations that they observed. They should provide a complete table and list all mutations observed in the sequenced clones.

**Response:** All this information was shown in Fig. 1d.

**Reviewer #3:**

The manuscript, “Global mistranslation increases cell survival under stress in E. Coli” demonstrates that mistranslation brings cells closer to the threshold level of misfolded proteins required for induction of a stress response. This in turn helps cells survive in stressful environments. This is an exciting finding in the field of evolutionary genetics and cell biology, and has important implications related to medicine and aging. The authors for the most part describe a logical progression through the experiments and how they reached this conclusion. The conclusion is justified by the data.

**Response:** We thank the reviewer for their encouraging comments.

**Comment 1a:** I do have a few major concerns that need to be addressed.
Major concern 1: does evolution maintain high mistranslation rates globally just because they are beneficial in some stressful environments?
a. The abstract starts by asking why global mistranslation rates are high. I am not sure this manuscript addresses that question. It shows why increased mistranslation rates are beneficial in stressful environments. It does not show they are generally beneficial. Thus, it seems unlikely that evolution maintains high mistranslation rates globally just because they are beneficial in some environments. More problematically, I am not sure the authors are even arguing that this is true. Can they authors make a more explicit statement about whether their results suggest that natural selection maintains high mistranslation rates globally due to the benefit this provides in stressful environments?

**Response**: Thank you for pointing this out. We do not directly show that mistranslation rates are generally beneficial, or that the fitness advantage it confers under stress is sufficient to drive an increase in general mistranslation rates. However, the mechanism underlying the advantage of mistranslation – early activation of the SOS response – allows us to speculate that mistranslation may be broadly beneficial under multiple stresses, and may evolve under positive selection in some conditions; e.g. if cell frequently encounter stresses (e.g. DNA damage) where the SOS response is critical. As discussed in the text (Page 10, lines 307-310), most agents that damage DNA also damage proteins, and can lead to mistranslation; therefore, it seems intuitive for the two to be linked. Hence, our results make it plausible that a generalized benefit of mistranslation during stress could allow the evolution of higher baseline mistranslation rates. Of course, more work is required to actually demonstrate this.

Please also see our response to Reviewer 1, who raised a related issue. We hope that our interpretation of the results is now clearer.

C**omment 1b**. The authors test 49 different environments using Biolog, but only report results from a few in figure 1. What about the other environments? Is mistranslation usually beneficial or deleterious? I bet it is usually deleterious, making it difficult to argue that maintaining high mistranslation rates is adaptive.

**Response:** Please see our response to Reviewer 2’s comment 1, addressing this issue.

**Comment 1c.** How come cells don’t just decrease the threshold level required to activate stress responses? Wouldn’t that be more efficient than maintaining sloppy protein synthesis and all of these misfolded proteins?

**Response:** In our mistranslating strains, rapid SOS activation is a by-product of mistranslation. We do not propose that high mistranslation rates evolve primarily to activate stress responses. Instead, we propose that the large benefit of mistranslation under diverse stresses (that can be mitigated via the SOS response) may weaken selection against high mistranslation, particularly if the stress is encountered frequently or is highly deleterious.

**Comment 2**: Some of the earlier results and experiments are not described in enough detail.
a. See point 1b above. More information is required about these initial experiments including whether the wt strain did better in most environments, and how many replicates were performed.
b. The results section says the mutant outperformed the wt in ‘many’ of the DNA-damaging agents. How many? Which ones? Why not all?
c. Figure 1 shows significant differences between mutant and wt in a subset of conditions. Are the magnitude of these differences very large compared to the way the wt and mutant grow in other conditions? Does this significance account for multiple hypothesis testing (i.e. the fact that 49 experiments were run)? We need to see a summary of the other conditions, or a comment about this is the main text.
d. All of these things should be discussed in the main text in the results section. Including experimental details in the introduction and a ‘see introduction’ in the results section is atypical.

**Response:** We have now addressed the issues with the Biolog screen, as noted in our response to Comment 1 by Reviewer 2. We have summarized the Biolog preliminary results in the results section (Page 5, lines 133-136) and provided more details in Fig S1.

The sentence mentioned in point (b) above referred to data shown in Fig 1, for the three kinds of DNA damage that we tested in more detail, and not to the Biolog results. We have now clarified this, replacing “many” with “three” (line 137).

In point (d) above, the ‘See Introduction’ referred to the Mutant strain and the fact that it carries less initiator tRNA than the WT; not to the Biolog results. We have now rephrased the sentence, so this confusion should not arise.

**Comment 3:** Explain why stress responses are not activated all of the time.
a. After reading the paragraph of the introduction including line 85, the immediate response is to question why stress responses are not activated all of the time. The reason is that activating these responses slows growth rate a little because these responses are costly to manifest. So it makes sense to activate them only when the situation calls for it, i.e. in stressful environments. Given this manuscript discusses the threshold level of stress that is required to activate these responses, this information needs to be made clearer in the introduction. As written, this section of the introduction is not only confusing, it is also a missed opportunity to get the reader thinking about the idea that organisms make decisions about when to activate their stress responses and these thresholds may differ for different organisms, cells or mutants.

**Response:** We agree and thank the reviewer for pointing this out. We have added a line to this effect in the introduction (Page 4, lines 82-84) and also brought it up in the discussion (Page 11-12, ines 367-375).

**Comment 4.** Improve figure 2C. The circles in a grid imply 96-well plates, or 24-well plates, or some kind of manufactured piece of equipment. If you mean each circle is a cell in a population, perhaps do not place these into a perfect grid. This figure should be larger and more detailed since it is your main hypothesis.

**Response:** Thank you, we have made the change (Fig.2c).
