# Supplementary material for: Global mistranslation increases cell survival under stress in Escherichia coli
Source: PLoS Genet. 2020 Mar 9;16(3):e1008654. doi: 10.1371/journal.pgen.1008654 (PMC7082066; doi:10.1371/journal.pgen.1008654)
Supplement: Attachment — Submitted filename: Response to reviewers 2.docx [file pgen.1008654.s019.docx]

**Response to reviewers**

**Reviewer #1:** I am not satisfied with the authors’ response to my previous concern that the writing is unclear about the distinction between baseline mistranslation rates and stress-induced mistranslation rates. In their response, the authors point to lines 55-58 as clarifying that they wish to hypothesize bet-hedging advantages even with respect to baseline rates. But those lines make little sense. Do the authors mean that cells elevate mistranslation above basal rates, rather than that “cells elevate basal mistranslation”, which would seem to me to be an oxymoron? If not, then I cannot parse the sentence (eg do they mean that evolution elevates basal mistranslation?) If they do mean that cells elevate mistranslation above basal rates, then I do not see how this addition makes the hypotheses applicable to baseline levels, as the response to reviews says that it does. The cited data shows that stress-induced elevation actually does happen, and I feel that this fact counts strongly against the authors’ now-stronger-than-before interpretation of the findings of this manuscript as pertinent to baseline rates.

However, I continue to find the results and hence the manuscript as a whole to be novel, important, and convincing. It is a lost opportunity that the conceptualization and framing of the significance and implications of those results has not been improved.

Signed,
Joanna Masel

**Response:**

We are sorry that the distinction was unclear. Upon re-reading the text, we do see that it could be framed better. To eliminate ambiguity in interpretation, we have now clearly outlined the first two hypotheses to be instances of ‘baseline mistranslation’ (Line 56) and the third to be ‘stress-induced mistranslation’ (Line 58). The fourth example – statistical proteome – can and does occur in both basal and stress-induced mistranslation, and this has been added to the text (Lines 64-69). We did mean that cells elevate mistranslation above baseline (pre-existing) levels under some stresses (data cited); we hope that the current wording in the text clarifies our position.

We also realised that our use of the word ‘induced’ might be the source of some confusion here. We would like to clarify this. There are three kinds of mistranslation discussed in the text.

1. Baseline: The mistranslation levels that occur in cells as a result of biochemical limitations during decoding nucleotides into amino acids, possibly including a speed/accuracy tradeoff. The impact of these errors may be hidden from natural selection by mechanisms such as chaperones that effectively buffer the negative impacts of protein mistranslation (and therefore misfolding). Baseline mistranslation rate could potentially evolve under selection to be higher or lower (this is now discussed more clearly and explicitly in the introduction).
2. Induced: We had used this to indicate that we artificially ‘induce’ higher mistranslation in cells through genetic and environmental manipulation. Note that this induction was still “basal”, in the sense that at all times, the manipulated cells had greater-than-WT basal mistranslation (or lower, in the case of hyper-accurate ribosomes). However, to avoid confusion we now say “increase” or “decrease” instead of “induce” when referring to experimental manipulation.
3. Stress-induced: Stress- induced mistranslation is a special case where mistranslation is increased beyond pre-existing baseline levels upon exposure to a stress. For example, through oxidation of tRNAs leading to incorrect recognition of the tRNA during decoding (reviewed by Mohler and Ibba, 2017, cited in the text). In our manuscript, we do *not* present any evidence of stress-induced mistranslation: DNA damage and high temperature do not lead to elevated mistranslation. What we do find (now added in the text in the Discussion, Lines 319-324) is that when mistranslation levels are elevated (no matter how they are elevated), rapid SOS activation is a by-product, allowing rapid DNA repair. When we lower basal mistranslation (by making the strain hyper-accurate in decoding nucleotides to amino acids), this activation goes down, reducing survival under DNA damage.

To make this clearer, we have now used ‘baseline’ and ‘stress-induced’ in the text. It is also clarified that we are changing mistranslation levels by artificial manipulation (such that they are consistently higher or lower than the baseline) and not through exposure to stress.

**Reviewer #2:** The authors revised according to clarify all of my and other reviewers comments. I don't have any further concerns to publish this manuscript.

**Response:** We are glad that we could resolve the issues raised. We thank Reviewer 2 for their comments.

**Reviewer #3:** The authors have addressed my previous concerns about the clarity and lack of detail in the description of the methods and data.

**Response:** We are glad that we could resolve the issues raised. We thank Reviewer 3 for their comments.

**Reviewer #3, Comment 2:** A concern that remains is about their hypothesis that there is a “broad fitness benefit of global mistranslation”. The authors frame their paper around this idea, but I am concerned that it is not supported by the data.

The authors show that mistranslation is beneficial in certain environments because it activates cellular stress responses. The link between mistranslation and activation of these responses is interesting. Why not focus the paper there, closer to the data. The argument that this link underlies the baseline mistranslation rates observed across the tree of life is far reaching.

Firstly, understanding whether a trait that is only beneficial in certain environments will be maintained by selection is a complicated question that this paper does not address. It is indeed a difficult question to address because it requires knowledge of how often those environments are encountered, and the selection coefficients across all environments.

Secondly, if activating stress responses is generally a good thing, why wouldn’t these responses evolve such that their baseline level of expression was slightly higher. Thus, mistranslation would not be required to achieve the ideal level of activation.

All reviewers felt that the authors decision to focus on the global fitness benefits of mistranslation was not consistent with their data. Reviewer one asked the authors to separate the evolution of baseline mistranslation rates from the evolution of these rates in high stress environments. Reviewer two writes, “I feel that the argument that the author made in the manuscript “mistranslation can be adaptive trait” is overstating.” I agree with both other reviewers in that the data presented in this manuscript do not tell us anything definitive about the evolution of baseline mutation rates. Nonetheless, I think these data are interesting and the paper should be re-framed to talk about the link between mistranslation and cellular responses to stress. The discussion about how global mistranslation rates evolve could be included in the discussion but should not drive the paper.

**Response:** We made some changes previously in this regard in response to Reviewer 2’s comments. For instance, we no longer use the line ‘Mistranslation can be an adaptive trait’. We agree that we do not present any evidence for the evolution of mistranslation rates, either baseline or stress-induced. However, we do present evidence that whatever baseline mistranslation rates exist are important for activating the DNA damage response and contribute to cellular survival under this stress (because lowering basal rates by making ribosomes hyper-accurate reduces survival under DNA damage).As suggested, we have now restricted speculation about the evolution of mistranslation rates to the discussion section, and we do not talk of a broad general advantage to mistranslation (Please see Discussion lines 338-340). Regarding the comment ‘Mistranslation would not be required..if baseline SOS expression was higher’ please see our response to Reviewer 1, and also Discussion lines 319-324, and 383-396
